# Clothing and Health
**The Influence of Clothing on Health*. By Fred. Treeves, F.R.C.S., etc. Cassell and Co.


**Published:** 1887-01-29

**Authors:** 


### Clothing and Health.*

He who attempts to reform the civilised method of clothing undertakes a labour, in comparison with which the classical exploits of Hercules are but as child's play The God of the Club did battle with the physical, the material, something which could be knocked upon the head ; the clothes reformer, however, has a more subtle and more powerful enemy, the Evil Spirit of humanity ; for with sin came clothes into the world, and ever since our first parents acknowledged their transgression by stitching a few fig-leaves together, the moral and material garments have been closely related. As sins have increased in number and complexity, so have garments, till the complications of the one can be only equalled by the complications of the other, and the enumeration and description of them would occupy a large fraction of eternity.

We know it is possible, though difficult, to convert a sinner into a healthy moral being ; but, can we convert a dandy or a fashionable lady into a sensible dresser ? Alas, there is much more hope for the sinner. Has that great apostle of moral clothing, Teufelsdrockh, by all his erratic and eccentric preaching reduced the number of dandies by one? We fear not. If, then, the Great Prophet of modern times has failed, how can the minor prophets hope to succeed ? How, indeed ! Still, as our Clothes Bible says, "to us also is given, if not victory, yet the consciousness of battle, and the resolve to persevere therein while life or faculty is left." We therefore welcome this little book of Mr. Treeves as another advance of the army of sense and righteousness against the army of stupidity and sin.

Mr. Treeves is the preacher of facts ; he tells us what to do, and how to do it, and leaves the rest to Conscience and to Sense—two very feeble powers in this age of education. A few there may be who, honestly seeking the truth, when they have read this book will take to themselves woollen underclothing, and the other paraphernalia of hygienic dress ; but these will be few indeed. The majority will read the book doubtless with interest, will quite agree with Treeves, and will continue on the broad road which leadeth to disease ; strongly condemning, however, those follies from which they themselves are free, they thus

> "Compound for sin they are inclined to
> By damning those they have no mind to."

That a lady, the mother of a family, should wear woollen underclothing when it adds one-sixth of an inch to the circumference of her waist, is a ridiculous proposition. We say : "Certainly not, dear lady ; the thing is absurd ; but your husband and your children. Don't you think they might do so with advantage ?" The "dear lady" may see sense in this, for we are all Spartans at bearing the afflictions of others, and are adepts at removing the mote from our neighbour's eye. In the matter of clothes, we would thus try to divide the house against itself, setting mother against daughter, daughter against mother, husband against wife, and wife against husband,—hoping that from the abundance of preachers some converts may be made. The difficulties before the Clothes Reformer are enormous ; indeed, to get women to wear the best hygienic dress, divided skirts, etc., is, we fear, impossible. The young and elegant no doubt would, they may wear what they please, looking well in all things : but, to get a woman of forty, weighing eighteen stone, to adopt divided skirts, etc., would be a task as difficult as undesirable.

Mr. Treeves treats of clothing simply from the hygienic point of view. We would like to see as able

* *The Influence of Clothing on Health.* By Fred. Treeves, F.R.C.S., etc. Cassell and Co.

a treatise on the Influence of Clothing on the Purse and Temper of Husbands—a most important subject, and well worthy of a rigorous diatribe.

We can recommend this book as an admirable little treatise, the subject being dealt with in a very thorough and interesting manner. It is well worth a most careful perusal by all: only reader, when you have read it, do not put the advice in the shelf along with the book. Put the advice into practice: wear wool, "purge, leave sack, and live cleanly."

